# Soft and Adhesive Cardiac Patch for Electrophysiological and Contraction Measurement

**DOI:** 10.1002/advs.75098

**Published:** 2026-04-07

**Authors:** Wuliang Chen, Xinyu Shen, Hanning Liu, Lamei Du, Caicai Jiao, Longfei Li, Qian Wang, Qiuting Zhang, Lixue Tang, Liang Hu, Yubo Fan

**Affiliations:** ^1^ School of Biological Science and Medical Engineering Beihang University Beijing P. R. China; ^2^ School of Biomedical Engineering Capital Medical University Beijing P. R. China; ^3^ National Center for Cardiovascular Diseases National Clinical Research Center for Cardiovascular Diseases State Key Laboratory of Cardiovascular Disease Department of Cardiovascular Surgery Fuwai Hospital Key Laboratory of Coronary Heart Disease Risk Prediction and Precision Therapy Chinese Academy of Medical Sciences and Peking Union Medical College Beijing P. R. China; ^4^ School of Mechanical Engineering and Automation Beihang University Beijing P. R. China; ^5^ College of Automation Engineering Northeast Electric Power University Jilin P. R. China

**Keywords:** high conformability, implantable epicardial electronic patch, liquid metal, low Young's modulus, reliable adhesion

## Abstract

Epicardial patches have been developed for real‐time monitoring of the normal electrophysiological activities of the heart. However, the modulus of most patches is much higher than that of the heart, which can potentially be stripped from cardio surface and impose limitations on cardiac function. Here, an ultrasoft and adhesive cardiac patch is proposed to achieve high conformability to the beating heart surface owning to the sophisticated multi‐material assembly process of each ultrathin and soft layer, resulting low Young's modulus close to that of the heart (∼ 65 kPa) and favorable tissue‐adhesion properties (∼ 27 N m^−1^) due to the hydrogen bond network provided by tannic acid and the covalent binding of carboxyl groups to amino groups. In addition, this patch can not only record the electrophysiological activities of the epicardium, but also measure contraction force of the myocardium, which provides a new monitoring perspective for the diagnosis of heart diseases. Therefore, it is believed that this epicardial electronic patch has great potential in fields such as ischemic heart disease.

## Graphical abstract

This study developed an epicardial electronic patch with a modulus similar to that of the heart (approximately 65kPa), which can collect stable electrocardiogram signals and myocardial contractility on the surface of a dynamically beating heart. This is attributed to the hydrogen bond interaction formed between the amino and catechol groups in the hydrogel and the tissue surface.

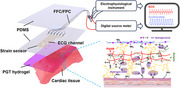

## 1 Introduction

The rapid advancement of soft bioelectronics has facilitated the emergence of a new generation of high‐performance implantable epicardial electronic devices. These systems are designed to form device–tissue interfaces that maintain long‐term stability and remain nearly “mechanically invisible” during the heart's intense and cyclic contractile–relaxation motions [1, 2, 3]. The realization of such an interface is essential for acquiring high‐fidelity electrophysiological signals free from motion artifacts, as well as for enabling precise monitoring and treatment of cardiac diseases such as arrhythmias. An ideal epicardial electronic device should fulfill the following requirements: i) Its mechanical properties—particularly the elastic modulus—must conform perfectly to and match the heart surface; ii) Unlike traditional invasive approaches such as suturing, it should achieve rapid and instantaneous adhesion to the moist, dynamically active cardiac surface, thereby minimizing tissue damage as much as possible; iii) It must exhibit excellent fatigue resistance and self‐healing capabilities, enabling it to withstand repeated stretching cycles without failure while recording signals with a high signal‐to‐noise ratio.

To address the aforementioned requirements, recent research efforts have primarily focused on two strategic directions: structural engineering optimization [4, 5, 6, 7, 8] and material innovation [9, 10, 11, 12]. The former often employs wavy, serpentine, or fractal architectures to interconnect rigid yet high‐performance inorganic electronic components (such as silicon nanomembranes and metals), strategically placing them near the device's “neutral mechanical plane” to dissipate stress through structural deformation under tensile strain [4]. However, these “rigid‐island” components often fail to conform fully to tissue‐level deformations during cardiac cycles at the microscale, and their inherent stiffness may even induce unintended mechanical constraints on the myocardium. On the other hand, material‐based strategies aim to develop intrinsically stretchable materials that improve modulus matching and enhance tissue compliance. For example, core–shell structured silver nanowires coated with gold have been embedded within elastomeric polymers to form conductive composites used in epicardial mesh electrodes [9]. Additionally, active multiplexed arrays based on silver nanowire/polymer composites have been developed for high‐resolution monitoring of cardiac activity [10]. It is noteworthy, however, that although intrinsically stretchable nanocomposites incorporating solid conductive fillers can achieve bidirectional adhesion to the epicardium, the high loading of rigid fillers and the use of low‐toughness matrix polymers often lead to fatigue fracture under cyclic loading, ultimately compromising device longevity. Moreover, current fixation methods for such devices still rely heavily on surgical suturing or commercially available bioadhesives. These approaches carry inherent invasiveness and potential bioincompatibility, which may impose subtle mechanical restrictions and cause chronic damage to the target organs under monitoring [3, 13, 14].

**FIGURE 1:**
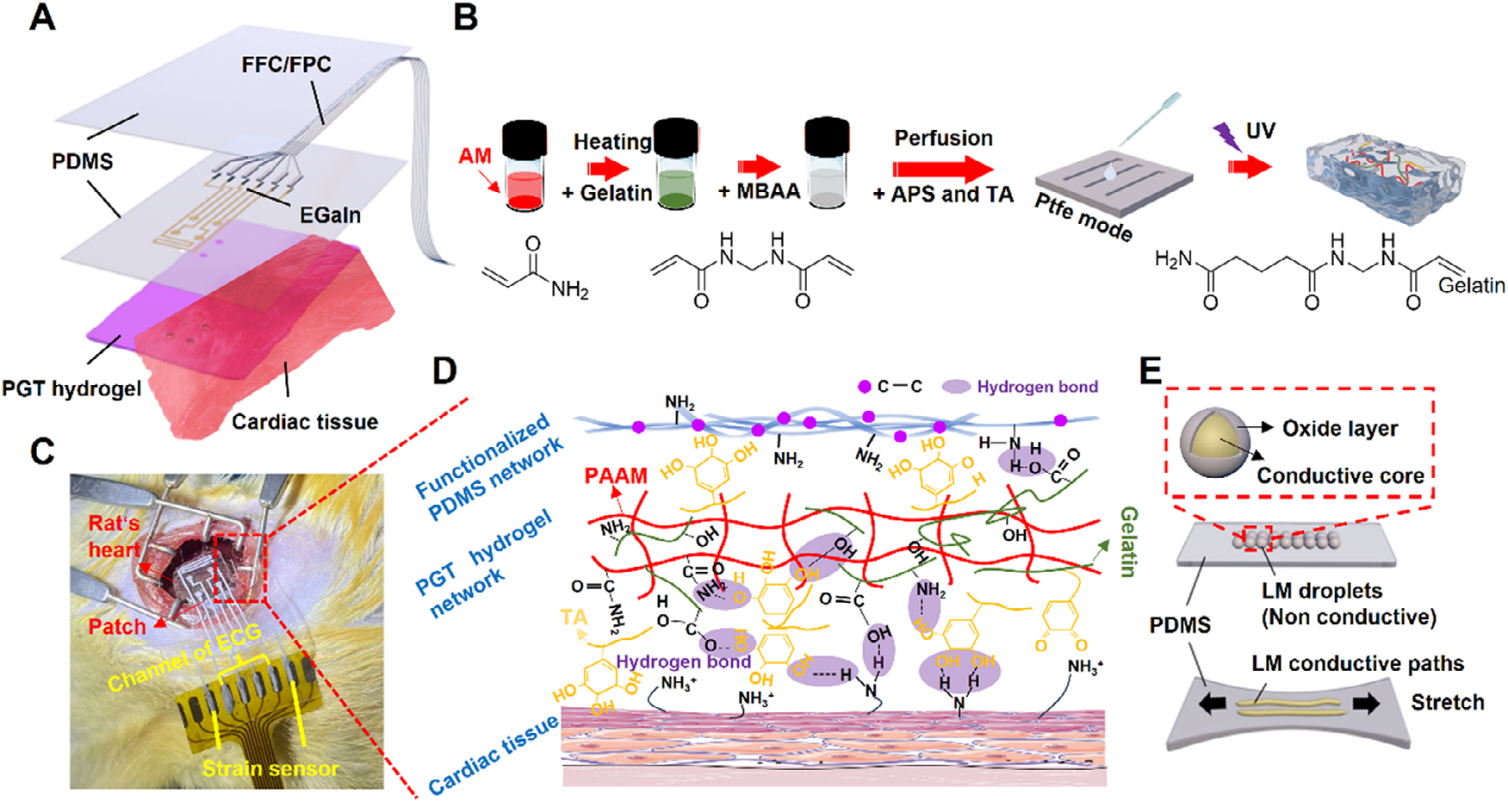
(A) Schematic layout of an implantable epicardial electronic patch. (B) The preparation process of PGT hydrogel. Acrylamide and gelatin were dissolved in deionized water, and tannic acid solution was added to increase the gel adhesion. Then, the precursor solution with crosslinking agent and initiator was injected into the mold to form hydrogel structure under ultraviolet light. (C) The patch adheres to the epicardium for detecting cardiac strain and ECG signals. (D) The mechanism of double‐sided adhesion of hydrogels results from the bonding between carboxyl groups in the hydrogel and amino groups at the interface, as well as the hydrogen bond network formed by TA. (E) The formation mechanism of conductive pathways. The liquid metal particles are subjected to force, causing the oxide layer to crack and exposing the conductive core.

To systematically address the fundamental challenges in material properties, interfacial adhesion, and integration strategies outlined above, this study presents a low‐cost fabrication method for an epicardial electronic patch with a sandwich‐style architecture, as illustrated in Figure 1A. The patch consists of: an interfacial layer composed of polyacrylamide/gelatin/tannic acid (PGT) hydrogel, which offers excellent adhesion and biocompatibility for direct contact with cardiac tissue; ultra‐flexible interconnects formed using eutectic gallium–indium (EGaIn) liquid metal (LM), which minimally influences the overall mechanical behavior; and an encapsulation layer of elastomer PDMS that provides mechanical stability and insulation. By carefully tuning the mechanical properties of the elastomer with low polymerization (30:1) (Figure S1) and surface modifications, the resulting the whole patch with multilayers exhibits an extremely low Young's modulus (∼ 65 kPa)—closely matching that of human tissue [14, 15]—along with reliable tissue adhesion (∼ 27 N m^−1^), enabling conformal attachment to the surface of a rat heart (Figure 1C). Notably, the patch maintains strong adhesion without delamination during cyclic stretching owing to the muscle‐inspired molecular design of the hydrogel, which incorporates tannic acid and a gelatin network (Figure 1D). The abundant catechol groups in tannic acid facilitate the formation of a hydrogen‐bonding network with substrate surfaces, significantly enhancing adhesion strength. Simultaneously, the gelatin network acts as an energy‐dissipative matrix [16, 17], effectively distributing stress through reversible chain alignment, sliding, and reorganization under load, thereby suppressing interfacial crack propagation and ensuring adhesion durability and resistance to peeling during repeated deformation. Furthermore, when the liquid metal droplets embedded in the PDMS are subjected to stretching, the rupture of their surface oxide layers enables the formation of continuous conductive pathways (Figure 1E). This dynamic and liquid‐based reconnection mechanism mitigates fatigue‐related failures commonly encountered in cardiac monitoring, thereby enhancing device reliability and operational lifetime [18, 19, 20]. Both in vitro and in vivo experiments confirm that our epicardial electronic patch achieves high conformability on the beating heart and enables continuous electrophysiological recording and mapping. This integrated material and interface design strategy offers a promising avenue for the development of next‐generation, long‐term stable bioelectronic adhesives with broad application potential.

## 2 Results and Discussion

### 2.1 Preparation and Characterization of PGT Hydrogel

We prepared the PGT hydrogel via a one‐pot method (Figure 1B). Specifically, a certain amount of acrylamide and gelatin was added to deionized water and kept at 60°C until the gelatin was completely dissolved, resulting in a homogeneous mixed solution. Subsequently, N,N'‐methylene bisacrylamide (MBAA) and ammonium persulfate (APS) were added to the above mixture. To enhance the hydrogel's adhesion to wet tissues, tannic acid (TA) was also incorporated into the mixed solution before UV crosslinking. The prepared hydrogel precursor solution was then injected into a mold and exposed to ultraviolet light. During this process, the gelatin network and the Polyacrylamide (PAAM) network formed physical entanglements, successfully synthesizing the PGT hydrogel. The physically entangled PGT hydrogel exhibited high stretchability (Figure S2). Benefiting from the temperature‐dependent sol‐gel transition properties of gelatin, the stretched PGT hydrogel could recover its internally broken gelatin chains through heating or cooling, thus demonstrating excellent stability and stretchability. Furthermore, through the chemical cross‐linking method, acrylamide can provide a permanent and elastic structural framework, ensuring the shape of the PGT hydrogel and preventing the collapse of the structure under physiological conditions. Numerous studies have been conducted on interpenetrating network (IPN) hydrogels. For example, Sun et al. [21] fabricated an IPN hydrogel using alginate and divalent metal ions (Ca^2^^+^). Although the ionically crosslinked hydrogel was also tough and stretchable, it lacked the adhesive properties of the PGT hydrogel. As shown in Figure S3, the prepared PGT hydrogel can adhere closely to different materials.

**FIGURE 2:**
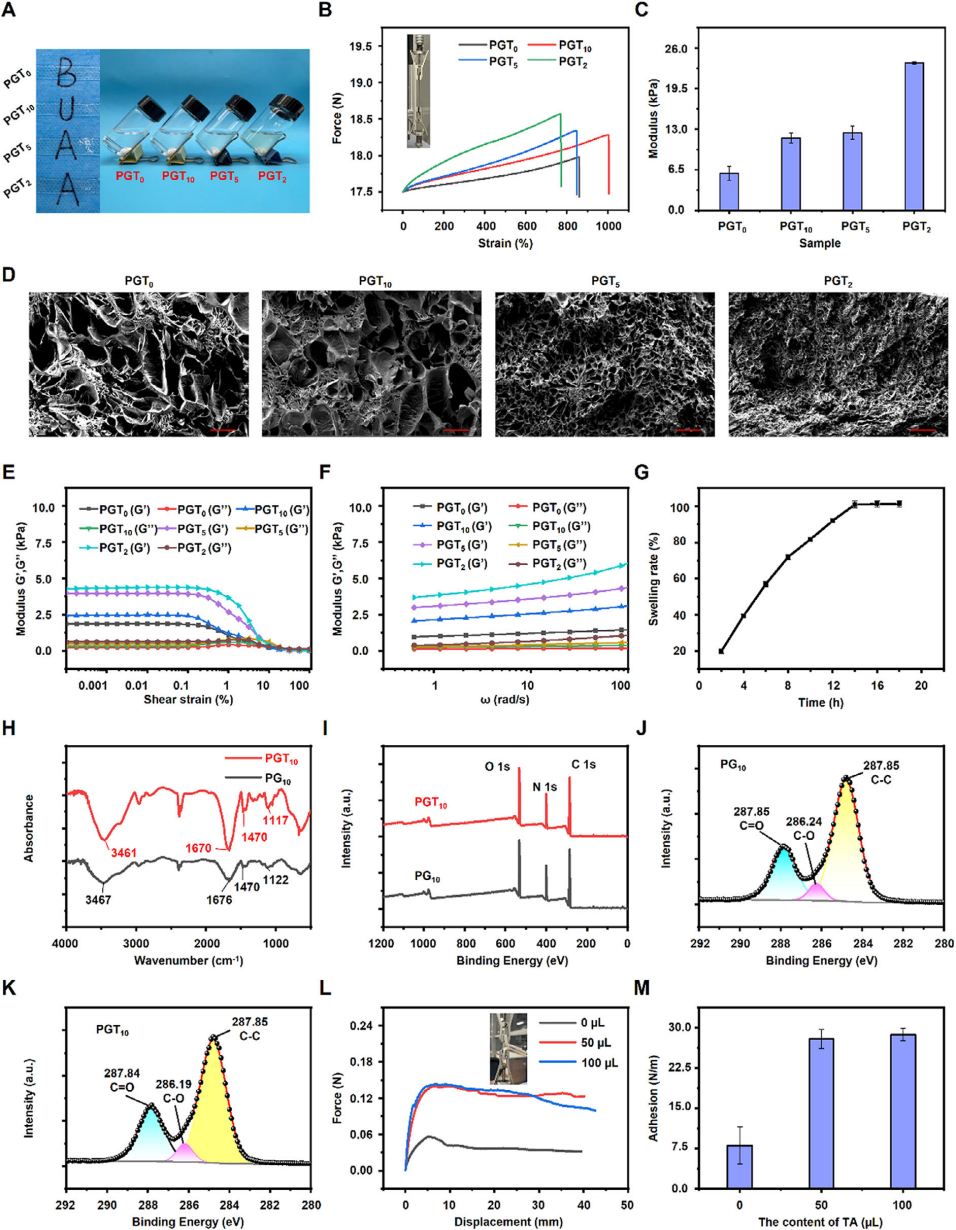
(A) Comparison of the transparency of PGT hydrogels with different ratios of acrylamide to gelatin and a schematic diagram of the uniformly mixed precursor solution used for synthesis. (B) Tensile property curve of PGT hydrogels with different ratios of acrylamide to gelatin. The schematic diagram of the tensile state of PGT_10_ hydrogels is shown. (C) Young's modulus of PGT hydrogels with different ratios of acrylamide to gelatin (*n* = 3). (D) SEM images of PGT hydrogels with different ratios of acrylamide to gelatin, scale bar: 50 µm. (E,F) Storage modulus (G') and loss modulus (G'') of PGT hydrogels in (E) amplitude sweep and (F) frequency sweep tests. (G) Swelling performance test of PGT_10_ hydrogels (*n* = 5). (H) Fourier transform infrared (FTIR) spectra of PG_10_ and PGT_10_ samples. (I) XPS survey spectra of the PG_10_ and PGT_10_ hydrogels. C 1s XPS spectra of the (J) PG_10_ and (K) PGT_10_ hydrogels. (L)180° peeling test of PGT_10_ hydrogels and PG_10_ hydrogels. (M) Adhesive strength of PGT_10_ hydrogels and PG_10_ hydrogels under peeling test (*n* =).

For bio‐adhesives applied to a beating heart, certain mechanical strength is essential. To this end, we characterized and conducted mechanical property tests on the synthesized PGT hydrogels. As shown in Experimental Section Table 1 (PGT_0_, PGT_10_, PGT_5,_ and PGT_2_ groups), this study synthesized four groups of hydrogels with different ratios of acrylamide to gelatin to investigate their characteristics. The four groups of synthesized hydrogels are shown in Figure 2A. As can be seen from the Figure 2A and Figure S4, with the increase of gelatin content, the transparency of the hydrogel slightly decreases. Then, the mechanical properties of the hydrogels with varying acrylamide and gelatin ratios are illustrated in Figure 2B,C. Compared to the pure PGT_0_ hydrogel, the addition of an appropriate amount of gelatin (PGT_10_) improved the mechanical properties of the hydrogel to some extent, with the elongation at break increasing from 860% to 1020% and the Young's modulus rising from 5 to 10 kPa. Gelatin molecular chains contain a large number of hydrophilic groups, such as hydroxyl, amino, and carboxyl groups, which interact with PAAM through hydrogen bonding and other interactions, contributing to the formation of the hydrogel's 3D network structure and thereby enhancing its mechanical properties. Furthermore, the gelatin network acts like an energy dissipator. When subjected to external forces, the molecular chains within it will “give way” and “reassemble” through reversibly oriented adjustment, sliding, and reconstruction, thereby ingeniously dispersible and absorbing stress. Therefore, compared with the pure PAAM network without gelatin, it has stronger tensile strength and a higher elongation at break. However, as the gelatin content continued to increase, the hydrogel's network structure became overly dense, hindering the diffusion and transport of water molecules and significantly reducing its tensile properties. SEM images (Figure 2D) further confirmed this phenomenon. The results in Figure 2D indicate that the pore area of the hydrogel is primarily determined by the PAAM network, but the increase in gelatin content led to a reduction in pore area. As a secondary network, gelatin ultimately influenced the mechanical performance of the hydrogel. In addition, this study also characterized and tested the mechanical properties of hydrogels without tannic acid (PG_10_). As can be seen from Figure S5A, compared with PG_10_ hydrogel, the pore structure of PGT_10_ hydrogel is slightly denser, which is consistent with the relevant literature reports [22]. The addition of tannic acid will form physical cross‐links with gelatin molecules. It can be seen from the mechanical tensile test (as shown in Figure S5B) that the modulus and elongation at break of PGT_10_ hydrogel are also slightly higher than those of PG_10_ hydrogel, demonstrating better mechanical tensile properties.

**TABLE 1:** Different ratios of PAAM to gelatin.

	AM(g)	Gelatin(g)	TA(µL)	DI water(g)
PGT_0_	1.27	0	50	8.730
PGT_10_	1.27	0.127	50	8.603
PGT_5_	1.27	0.254	50	8.476
PGT_2_	1.27	0.635	50	8.095

Meanwhile, we further evaluated the mechanical strength of the hydrogels using a rotational rheometer (Figure 2E,F). For hydrogel samples with different gelatin contents, both the storage modulus (G') and loss modulus (G'') were frequency‐dependent. The storage modulus reflects the material's ability to store elastic energy, which demonstrates the material's rigid characteristics. The loss modulus reflects the material's ability to lose energy due to internal friction, demonstrating the material's viscous property. Across all tested frequencies (1 to 10 Hz), G' was greater than G'', confirming their gel‐like behavior and indicating that the material exhibits excellent resilience, enabling it to quickly recover its initial state upon the removal of external forces. This demonstrates favorable mechanical strength, making it highly suitable for dynamic, beating organs. Throughout the testing period, G' and G'' remained stable, highlighting the robust mechanical stability of the PGT hydrogels. Additionally, the results show that as the gelatin proportion increased, the storage modulus of the samples significantly rose, which is consistent with the trend observed in the Young's modulus. Based on the comprehensive mechanical performance data, we selected the hydrogel with an acrylamide‐to‐gelatin ratio of 10:1 for subsequent experiments.

Subsequently, the swelling behavior of the PGT_10_ hydrogel was tested to investigate its sensitivity to biological fluids in implantation scenarios. The swelling behavior of hydrogels is primarily determined by their internal cross‐linked network structure. The results in Figure 2G demonstrate that the prepared hydrogel exhibits a low swelling ratio, ensuring the dimensional stability of the gel‐based electronic devices during implantation and preventing any adverse effects on their mechanical properties. In addition, the adhesiveness of the hydrogel is also crucial. To enhance the hydrogel's adhesiveness, TA was introduced into the precursor solution. TA is a natural water‐soluble polyphenolic compound containing abundant pyrogallol and catechol groups, which facilitate the adhesion of hydrogels to the surfaces of different substances [23, 24]. Figure 2H shows the infrared spectra of the hydrogel before and after the addition of tannic acid. Analysis shows that the characteristic peaks of PG_10_ hydrogel include the stretching vibration of N‐H at 3467 cm^−1^, the stretching vibration of amide I with C═O bond at 1676 cm^−1^, the planar bending vibration of amide II and III with N‐H at 1470 and 1122 cm^−1^, and the stretching vibration of C‐H bond. After the addition of TA, the N‐H characteristic peak moved to 3461 cm^−1^, shifted toward the low wavenumber direction, and the area of the absorption peak increased, indicating that the addition of polyphenols can increase the number of hydrogen bonds in the sample. In addition, the absorption peak area of the amide I band also increased. This might be due to the oxidation of tannic acid to form quinone derivatives, generating a large number of C═O groups, which enhanced the signal strength of the amide I band. The position of the absorption peak in the amide II band remains unchanged, while the absorption peak in the amide III band shifts blue to 1117 cm^−1^, with an increase in peak area, indicating a certain increase in the number of hydrogen bonds. FTIR result analysis indicated that the addition of tannic acid increased the number of hydrogen bonds in the hydrogel, but no new functional groups were generated. In addition, we also conducted XPS characterization of PG_10_ and PGT_10_ hydrogels, and the results are shown in Figure 2I. Consistent with the infrared spectroscopy results, the wide‐scan X‐ray photoelectron spectroscopy (XPS) of the hydrogel revealed the presence of C, N, and O, while the addition of tannic acid did not introduce any redundant groups or elements. By comparing the C 1s spectrum of the two hydrogels, it can be found that the addition of tannic acid led to a lower binding energy of C═O and C─O, indicating a significant increase in hydrogen bonds (Figure 2J,K) [25]. Biological tissue surfaces contain nucleophilic agents, including amines, thiols, and hydroxyl groups. The catechol groups (including o‐quinone groups) in the hydrogel can form hydrogen bonds with these nucleophilic agents on tissue surfaces, thereby adhering to biological tissues [26]. We conducted a 180° peel test on the PGT_10_ hydrogel to measure its adhesive strength. The data in Figure 2L,M show that compared to hydrogel samples without tannic acid, the hydrogel exhibits significantly improved tissue adhesion, slightly surpassing that of commercially available cyanoacrylate adhesives [27], and comparable to that of polydopamine‐based gels also used for tight bonding of dynamic hearts [28, 29]. It is worth noting that further increasing the content of tannic acid in the water gel precursor solution will result in the formation of complex precipitates, making the solution cloudy and hindering the formation of a transparent and free‐of‐impurity water gel (Figure S6). In addition, the experiment also prepared a hydrogel without gelatin molecules and tested the magnitude of its adhesion force. The results are shown in the Figure S7. As can be seen from the figure, the hydrogel without gelatin molecules also has a relatively high adhesion force, and its magnitude is basically close to that of the PGT_10_ hydrogel. This indicates that gelatin molecules play a relatively low role in tissue adhesion, and the adhesion of the hydrogel is inseparable from the effect of tannic acid. Although hydrogels without gelatin molecules have a certain adhesive force, their tensile properties and modulus are relatively low, which have been specifically analyzed in the previous text. Finally, we verified the adhesion stability of the hydrogel through dynamic cyclic stretching. As can be seen from the Figure S8, during 2000 cycles of tensile stretching within the strain range of 0–10%, the hydrogel exhibited good adhesion to the pig skin, suggesting that the prepared hydrogel is suitable for dynamic beating organs and can maintain stable adhesion.

### 2.2 Preparation of the Epicardial Electronic Patch Containing PGT Hydrogel

**FIGURE 3:**
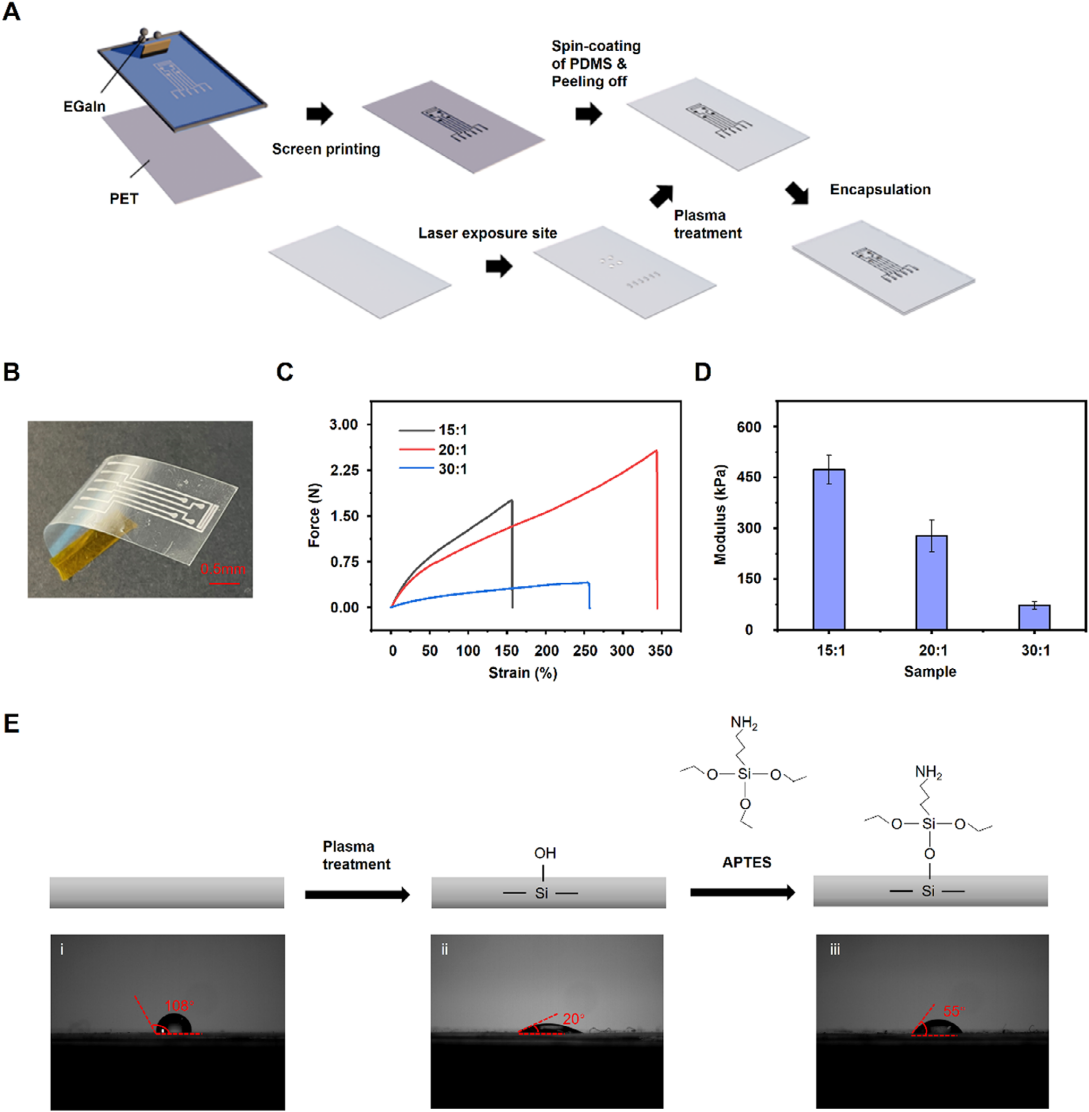
(A) Schematic diagram of the preparation of epicardial electronic patches. (B) A physical image of the epicardial electronic patch, with a thickness of approximately 100 µm and a high degree of flexibility, scale bar: 0.5 mm. (C) Tensile property curves of PDMS at different proportions. (D) Young's modulus of PDMS at different proportions (*n* = 3). (E) Functionalization of the PDMS surface (i) is the water contact angle of the unmodified PDMS film (ii) is the water contact Angle of the PDMS film after oxygen plasma treatment (iii) is the water contact angle of the PDMS film after APTES treatment.

Figure 3A shows the manufacturing process of the epicardial electronic patch, which begins with the construction of a liquid metal interconnection path on Polyethylene terephthalate (PET) substrate through screen printing. After the printed interconnection paths were placed in an oven at 80 °C to remove organic solvents, the PDMS prepolymer was spin‐coated onto the surface of the interconnection paths and cured at 70 °C for 2 h. When the cured PDMS is stripped off, the liquid metal interconnect path is transferred from the PET substrate to the PDMS, while at the same time, the liquid metal interconnect path is sintered under the action of shear stress (Figure 1E). Finally, through laser etching and plasma treatment, the encapsulation layer and the substrate layer are combined, and the electronic patch of the epicardium is prepared. The completed epicardial electronic patch is shown in Figure 3B, with a thickness of approximately 100 µm. The prepared epicardial electronic patch has high flexibility and can be stretched, bent, twisted, etc. (Figure S9). Among them, the liquid metal particle ink used for screen printing was prepared by an ultrasonic probe (Figure S10A). Using n‐decanol as the solvent, the large liquid metal droplets added were transformed into particles after being subjected to ultrasonic action for 5 min to obtain the liquid metal slurry. The average diameter of the liquid metal particles in the slurry is 2.5 µm (Figure S10B).

In order to reduce the Young's modulus of PDMS and make it softer to fit the tissue, some people have attempted to prepare PDMS elastomers by solvent‐thermal curing or adding chemical reagents. However, the extreme conditions required for the reaction or the unsafe reagents, such as N‐hexane, added to the prepolymer limit its expansion in the preparation method and its application in organisms. PDMS elastomers like Dow Corning 184 contain two components, namely double‐ended vinyl polydimethylsiloxane and hydrosiloxane. During the curing process, vinyl reacts with hydrosiloxane under the catalysis of a platinum catalyst to form a cross‐linked network as shown in Figure S1. Therefore, reducing the dosage of the crosslinking agent is the simplest and most effective way to lower the Young's modulus of PDMS. Here, the mechanical properties of PDMS under different crosslinking agent ratios (15:1, 20:1, 30:1) were experimentally tested, and the results are shown in Figure 3C,D. As can be seen from Figure 3D, when the ratio of PDMS prepolymer to crosslinking agent is 30:1, the Young's modulus of PDMS elastomer is 60 kPa, which is similar to the Young's modulus of mammalian heart tissue (∼80 kPa) [30]. Although further reducing the proportion of the curing agent helps to decrease the Young's modulus of the elastomer, the prepared PDMS film is not easy to form, and the increased adhesion force makes it difficult to peel off the substrate, which causes difficulties in the preparation of the device.

Nevertheless, the epicardial electronic patch prepared based on PDMS elastomer still adheres effectively to the tissue due to its chemical inertness [31]. Therefore, we used APTES to perform surface functionalization on the epicardial electronic patch. The water contact Angle experiment successfully proved the functionalization of the PDMS surface, and the results are shown in Figure 3E. Before being treated with oxygen plasma, PDMS has strong hydrophobicity, with a water contact angle of 108°. Subsequently, the PDMS treated with oxygen plasma had a water contact angle of approximately 20° due to the introduction of a large number of hydroxyl groups; Finally, APTES was used for treatment, and the water contact angle increased to approximately 55°. The alkoxy groups of APTES combine with the hydroxyl groups on the surface of PDMS through hydrolysis (one APTES molecule reacts with three hydroxyl groups). After the reaction, only one amino group in the APTES molecule is exposed, which is equivalent to three hydroxyl groups being replaced by one hydrophilic amino group. Therefore, the water contact Angle increases. By functionalizing the surface of the patch, that is, introducing amino groups into the surface of PDMS, and then pouring the hydrogel precursor solution onto the surface of PDMS and cross‐linking it under ultraviolet light, an epicardial electronic patch containing adhesive hydrogel PGT (PGT‐EEP) is finally obtained. To test the binding force between hydrogel and PDMS, the experiment adopted the shear lap method for testing, and the results are shown in Figure S11. It can be seen from the figure that the binding force between the hydrogel and PDMS is approximately 33.5 kPa. This indicates that the combination of PDMS and hydrogel has a high binding force due to the combination of amino groups with carboxyl groups in the hydrogel and the hydrogen bond network formed between tannic acid and the surface of the elastomer (Figure 1D), and also implies that the epicardial electronic devices prepared in this way will not produce delamination in applications, which is conducive to improving the quality of signal acquisition and the stability of the devices.

### 2.3 Performance Test and Characterization of PGT‐EEP

**FIGURE 4:**
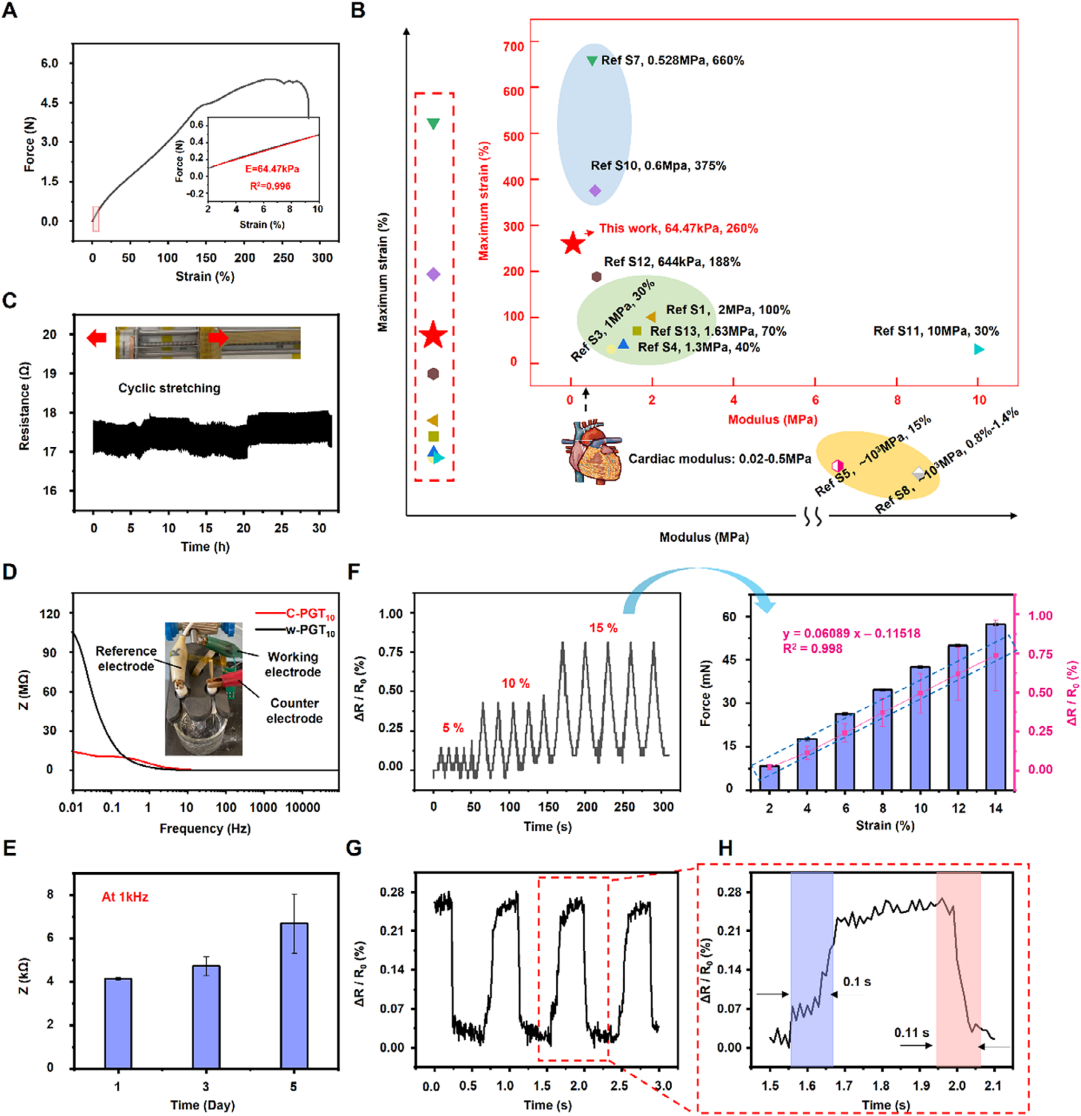
(A) Tensile property curve of PGT‐EEP. (B) The comparison of this work with other literatures in terms of modulus and stretchability. (C) The resistance variation curve of the liquid metal electrical signal conduction channel (length:35 mm; width:0.2 mm) under 10% strain. (D) Electrochemical impedance of patches without PGT_10_ hydrogel (w‐PGT_10_) and containing gel (c‐PGT_10_). (E) The impedance value of the liquid metal conductive paths in the PBS environment at 1 kHz (*n* = 3). (F) The resistance variation curves of strain sensor of PGT‐EEP under different strains (5%, 10%, and 15%) (left). The relationship between the resistance variation of PGT‐EEP and the force (right) (*n* = 3). (G) The resistance variation curve of strain sensor of PGT‐EEP in the hearts of beating pigs in vitro. (H) Response and recovery time of strain sensor of PGT‐EEP (*n* = 3).

Before being used for epicardial electrocardiogram monitoring, we first tested the mechanical and electromechanical performance of the PGT‐EEP sensor. For the tensile property curve, as shown in Figure 4A, the elongation at break of the PGT‐EEP sensor is basically consistent with that of the PDMS, and there is no significant change in Young's modulus either. We collected the relevant literature on epicardial electronic patches, and the results are shown in Figure 4B and Table S1. It can be seen from the table that the epicardial electronic patch designed and prepared in this study not only has high stretchability and extremely low Young's modulus, but also can be attached to the beating surface of the heart due to its own adhesion. The surface‐treated PDMS can be firmly combined with the hydrogel, avoiding the delamination of the substrate and the hydrogel. This greatly enhances the long‐term stable application potential of the pericardium electronic device and simplifies the steps in the experimental operation process, that is, it can be directly adhered without the need for external adhesives for auxiliary adhesion. This is also the difference between its inherent adhesion and other literature.

In addition, the operational lifespan represents a critical performance parameter of the device. To evaluate this characteristic, liquid metal conductive path was fabricated and subjected to cyclic tensile testing using a fatigue testing apparatus. As illustrated in Figure 4C, after 100000 cycles of tensile loading at a strain level of 10%, the resistance variation rate of the liquid metal remains as low as 1.7%, demonstrating the device's outstanding fatigue resistance.

For the acquisition of bioelectric signals, the size of the sensor impedance cannot be ignored. High impedance is the main obstacle to high‐fidelity recording of electrocardiogram signals due to significant signal attenuation and distortion. The impedance spectrum of the PGT‐EEP sensor obtained from the experiment is shown in Figure 4D. Compared with the electronic patch without PGT_10_ hydrogel, the impedance of the PGT‐EEP sensor is greatly reduced, which is related to the microstructure on the surface of the hydrogel. The surface of the liquid metal electrode is relatively smooth, so its effective specific surface area is limited. After the introduction of hydrogel, the complex and wrinkled micro‐surface greatly increased the specific surface area of the sensor in the physiological environment, thus the impedance decreased significantly. Furthermore, due to the presence of hydrogels, the direct effect of physiological fluids on the liquid metal electrode is avoided, which is conducive to the long‐term stable recording of the electrode [32, 33].

Meanwhile, the conductive path was placed in the PBS environment for five days to detect the changes in its impedance. As can be seen from Figure 4E, under the extreme condition of soaking for five days, although the impedance of the conductive path increases, it still has the ability to conduct electricity, which to some extent reflects its good stability. To more accurately describe its stability, we attached the electrodes before and after the immersion to the surface of the isolated pig heart that was subjected to the excitation signal and then collected the signals. The results are shown in Figure S12. The signals collected by the electrodes before soaking and after soaking for five days were basically consistent with the excitation signals, and their amplitudes and frequencies did not undergo significant changes.

Furthermore, we also tested the resistance changes of the strain sensor of PGT‐EEP under different strains (5%–15%, corresponding to the diastolic and systolic cycles of the heart [34]). The sensor demonstrated good repeatability at strains of 5%, 10% and 15% (Figure 4F, left), suggesting that it has great potential in sensing continuous cardiac diastolic and systolic cycles. We also established a connection between the force acting on the PGT‐EEP sensor under the same strain and the rate of resistance change, as shown in Figure 4F (right) and Figure S13. In this way, the resistance changes caused by the volume changes of the heart can be transformed into the magnitude of the force acting on the heart surface, presenting the force changes of the heart during the beating process more intuitively, which is conducive to the real‐time monitoring of the heart. Finally, in order to test the strain performance of the sensor more accurately, we attached the PGT‐EEP sensor to the surface of the beating pig heart outside the body (the beating of the pig heart is achieved by an air pump). As shown in Figure 4G, during the continuous beating of the heart outside the body, the sensor can withstand the continuous deformation of the heart and collect stable resistance change curves, demonstrating strong durability and repeatability, and has the possibility of long‐term monitoring of heart deformation. The resistance variation curve was analyzed, and it was found that the response time and recovery time of strain sensor of PGT‐EEP were 100 and 110 ms respectively (Figure 4H). Its rapid response and recovery speed play an important role in the real‐time monitoring process.

### 2.4 Biocompatibility and Longtime Properties of PGT‐EEP

**FIGURE 5:**
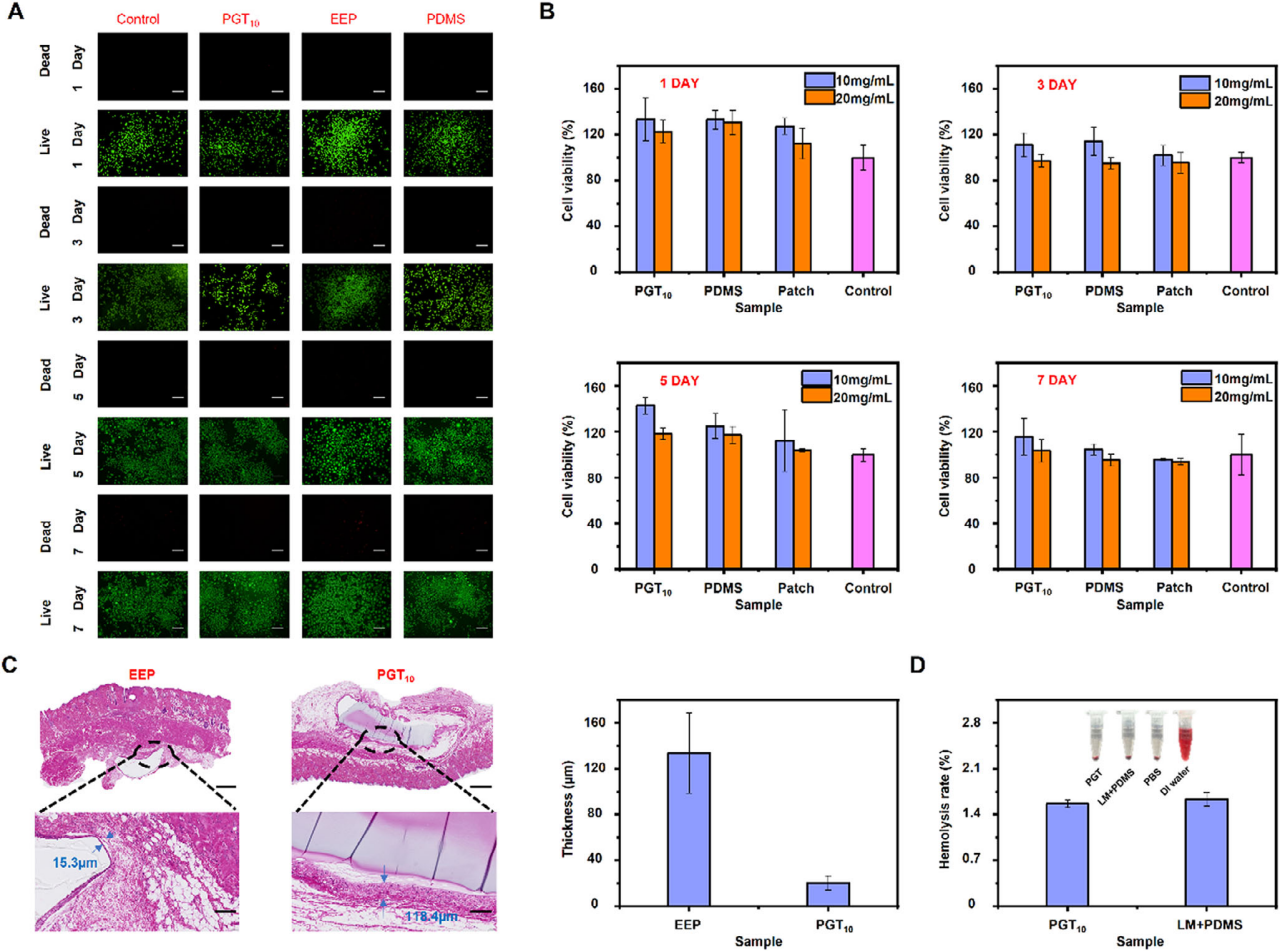
(A) Cell viability of fibroblast cells after 1, 3, 5, and 7 days incubation with different samples, including normal medium (control), PDMS, PGT_10_, and EEP, with a scale size of 100 µm. (B) CCK‐8 test results (*n* = 4). (C) The results of H&E staining of PGT_10_ hydrogel and the surrounding tissues of the patch, with a scale size of 200 µm and a scale size of 50 µm in the magnified area. Data in c (right) shows the measurement of the thickness of the subcutaneous fibrous sacs (*n* = 3). (D) Blood compatibility tests of different materials (*n* = 3).

Biocompatibility is of great significance in long‐term health monitoring. Therefore, we tested the cytotoxicity of PGT‐EEP through fibroblast cells. The fluorescence live/dead staining images show the state of the cells, with fewer dead cells (the staining result of propidium iodide (PI) shows only a few red dots), as shown in Figure 5A, which indicates that PGT‐EEP is safe and non‐toxic. Furthermore, the results of CCK8 also proved that PGT‐EEP has good biocompatibility (Figure 5B). It can be seen from the Figure 5B that after 7 days of culture, the survival rates of the cells were significantly higher than the 70% cytotoxicity threshold stipulated in ISO‐10993‐5. In addition, we also demonstrated the cytotoxicity of PGT‐EEP through the HL‐1 mouse cardiomyocyte cell line, as shown in Figure S14A. To identify cardiomyocytes, α ‐actin and 4′, 6‐diamino‐2‐phenylindole (DAPI) were stained to observe the morphology of cardiomyocytes (Figure S14B). What's more, in order to study the biocompatibility of PGT‐EEP in vivo, we implanted PGT‐EEP into the backs of rats for 7 and 14 days and then performed immunohistostaining (Figure 5C, left). The H&E results showed that PGT‐EEP had little effect on the surrounding tissues and no obvious inflammatory response (less recruitment and infiltration of inflammatory cells) occurred. We provide an enlarged view to clarify the form of the organization. We measured the thickness of the subcutaneous fibrous sacs of EEP and PGT_10_ (Figure 5C, right), which were approximately 133.73 ± 35.03 and 20.33 ± 6.15 µm respectively, meeting the standard of less than 150 µm in ISO 10993–6. Finally, we also collected fresh rat blood to verify the safety of PGT‐EEP, and the results are shown in Figure 5D. As can be seen from the figure, the solutions of the experimental group and the negative group remained uniform and transparent without any color change, suggesting that no hemolysis occurred. This is significantly different from the solutions of the positive control group. We further measured the absorbance of hemoglobin by microplate reader and found that the hemolysis rates of each material were all less than 2%, indicating that the electronic patches prepared from hydrogels, LM and PDMS did not cause damage to rat red blood cells.

### 2.5 In Vivo Animal Experiments

**FIGURE 6:**
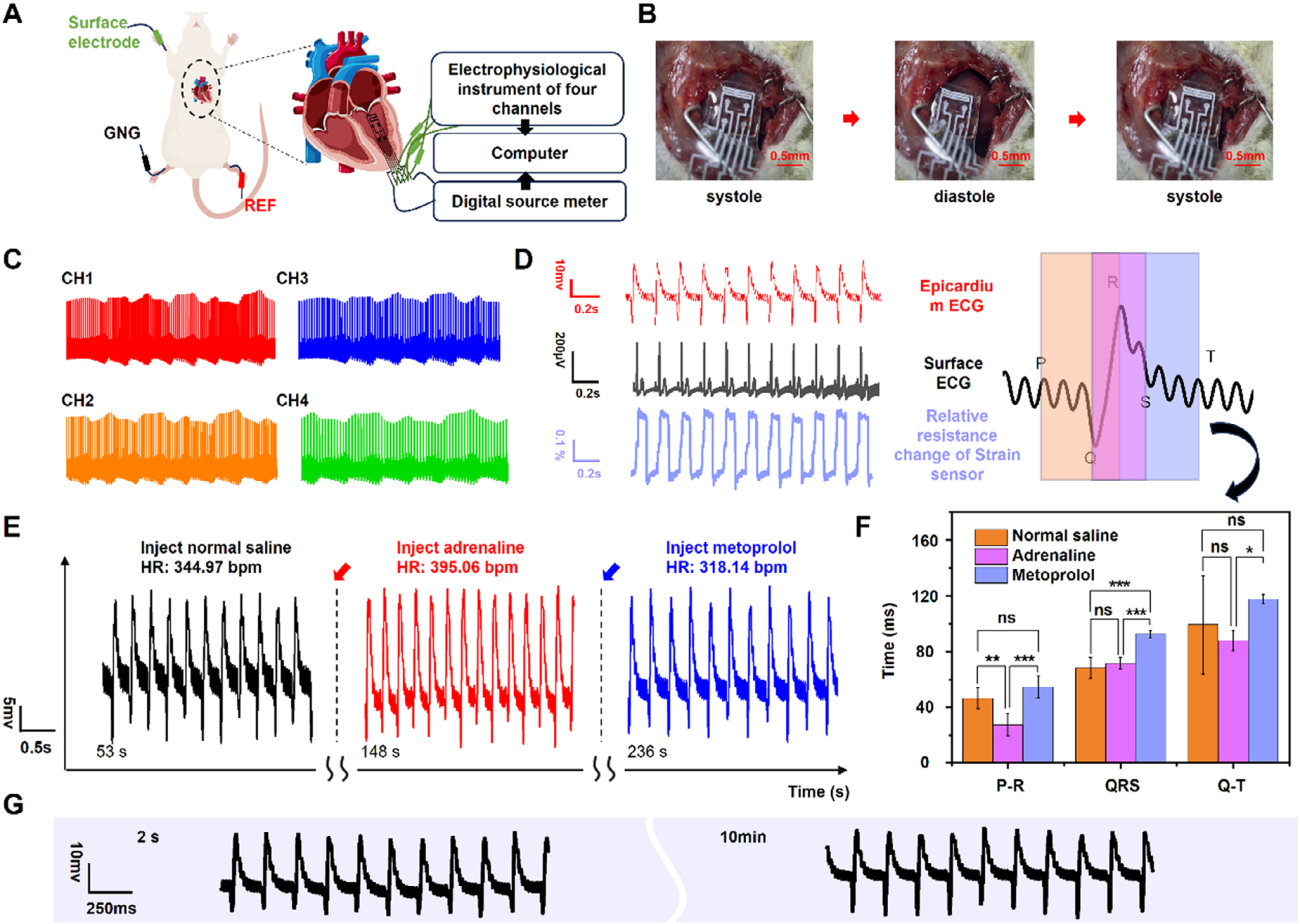
(A) Schematic diagram of the connection mode of PGT‐EEP or surface electrocardiogram. (B) Conformal attachment of PGT‐EEP to the right ventricle, scale bar: 0.5 mm. (C) Acquisition of epicardial electrocardiogram by four channels of the PGT‐EEP. (D) Comparison of epicardial electrical signals and relative resistance change of strain sensor obtained by PGT‐EEP with surface electrocardiogram. (E) Heart rate changes under the injection of normal saline, adrenaline and metoprolol. (F) The time of P‐R interval, QRS interval and Q‐T interval under the injection of normal saline, epinephrine and metoprolol (*n* = 5). One‐way analysis of variance (One‐way ANOVA) was used followed by Tukey's post‐hoc test to compare multiple groups, and the significance level was considered as ^*^ *p* < 0.05, ^**^ *P* < 0.01, ^***^ *P* < 0.001. (G) Representative electrograms after continuous recording of the epicardium for 10 min using PGT‐EEP.

PGT‐EEP was used to obtain the epicardial electrical signals of the beating hearts of rats and the mechanical signals during the process of cardiac volume changes, as shown in Figure 6A. The heart was exposed through thoracotomy at the third or fourth intercostal space on the left side of the rat, and the pericardium was removed with fine tweezers. The four channels of the PGT‐EEP was implanted on the surface of the ventricular epicardium, while the reference electrode and the auxiliary electrode were inserted into the left leg and the right leg respectively. In addition, the electrocardiogram signals on the body surface of rats were collected simultaneously in the experiment as controls. Figure 6B shows that the patch has a low Young's modulus and reliable adhesion. These characteristics ensure that the conproper contact during the diastolic and systolic cycles of the heart does not slide or delaminate (Movie S1). The results in Figure 6C indicate that the prepared PGT‐EEP can stably collect the electrocardiogram of the ventricular surface, and its frequency is consistent with that of the body surface electrocardiogram (Figure 6D). We analyzed the electrocardiogram signals collected by PGT‐EEP, and its signal‐to‐noise ratio (SNR) was approximately 38.38 dB. Furthermore, it can be seen from the figure that during the periodic variation process of the heart volume, the signals collected by the strain sensor also have good stability. We calibrated the resistance change and found that the force on the surface of the rat heart was approximately 0.02N. To further evaluate the performance of the electrodes, we injected drugs into rats to regulate changes in heart rate (HR). As can be seen from the Figure 6E, after the injection of epinephrine, the heart rate of the rats increased significantly, with an average heart rate of approximately 395.06 bpm. Subsequently, we injected metoprolol, and the heart rate of the rats decreased. At this time, the average heart rate was approximately 318.14 bpm. In addition, we conducted statistics on the P‐R interval, QRS interval and Q‐T interval of rats injected with different drugs, and the results are shown in the Figure 6F. After epinephrine injection, the P‐R interval and Q‐T interval were significantly shortened, which might be attributed to the increased atrioventricular node conduction velocity and the shortened effective refractory period. In addition, the QRS interval represents the depolarization process of the ventricular muscle, and the influence of adrenaline on it is limited, so its changes are not obvious. Conversely, after metoprolol injection, the P‐R interval, QRS interval and Q‐T interval all increased significantly, which was attributed to the reduction of myocardial excitability. From the above results, it can be seen that the prepared PGT‐EEP can still achieve stable electrocardiogram monitoring during the process of rapid heart rate changes. Finally, we conducted continuous epicardial recording of rats for 10 min (Figure 6G), involving more than 3000 diastolic and systolic cycles of the heart, which proved that the prepared PGT‐EEP had good durability.

## 3 Conclusion

In conclusion, we have proposed a manufacturing method for implantable epicardial electronic patches with low cost, excellent stability, reliable adhesion, and low Young's modulus. The patch has a Young's modulus similar to that of cardiac tissue and the hydrogel PGT on it has excellent tissue adhesion. The low modulus and high adhesion enable the patch to have conformal contact with the heart, greatly reducing mechanical differences. In addition, the liquid metal is confined within the elastomer, avoiding direct contact with biological fluids and ensuring the long‐term stability of the patch in the physiological environment. Finally, the designed electronic patch can not only collect the strain mechanical characteristics related to the diastolic and contrastive of the cardiac surface, but also establish multi‐channel electrographs, which is of great significance for revealing the physiological activities of the heart.

## 4 Experimental Section

### 4.1 Materials

Acrylamide, gelatin and tannic acid were purchased from Aladdin. All reagents were used as received. Milli‐Q deionized water was used in all experiments. The screen printing plates and PET were purchased from Taobao in China. PDMS kit (Sylgard TM 184) was purchased from Dow Corning Company.

### 4.2 Synthesis of PGT Hydrogels

Acrylamide and gelatin were mixed in a different ratio in deionized water, and the obtained mixture was stored in 60°C chambers and stirred. Table 1 shows four groups of the PGT hydrogel with different ratios of acrylamide to gelatin (PGT_0_, PGT_10_, PGT_5_, and PGT_2_ groups) that were used in this experiment. After the gelatin is completely dissolved and the solution is evenly mixed, ammonium persulfate (Sigma–Aldrich, 0.0059×acrylamide weight) was added as a photo initiator for acrylamide polymerization, and N,N‐methylene bisacrylamide (Sigma–Aldrich, 0.0006×acrylamide weight) was added as the corresponding cross‐linker. In order to increase the adhesion of the hydrogel, 50 µL of TA solution(5%)was also added to the mixture and cured for 30 min with UV.

### 4.3 Preparation of the Epicardial Electronic Patch

To prepare EGaIn inks for screen printing, 3 g eutectic gallium–indium alloy (Sigma–Aldrich, USA) and 500 µL n‐Decyl alcohol (Macklin, China) was added into a 2 mL EP tube for sonication. In order to prevent the evaporation of the solvent, the ink is ultrasonic for 5 min in an ice water bath. The morphology of EGaIn particles was characterized by SEM. The EGaIn particles inks were printed on PET substrates by a commercial screen printer. After screen printing, the pattern on PET substrates was baked at 80°C oven for 30 min to remove the residual solvent. PDMS prepolymer (Sylgard 184, Dow Corning) was spin‐coated on the top of the printed pattern (1 krpm, 10 s) and cured at 70°C oven for 3 h. After curing and peeling off, the conductive liquid metal pattern was transferred to PDMS substrates. Finally, through laser etching and plasma treatment, the encapsulation layer and the substrate layer are combined, and the electronic patch of the epicardium is prepared.

### 4.4 Surface Functionalization of the Epicardial Electronic Patch

To produce covalent coupling between epicardial electronic patch and PGT hydrogels interface, epicardial electronic patch was surface functionalized with primary amine groups. Specifically speaking, the patch was first activated or cleaned by oxygen plasma treatment applied for 3 min (30 W power) followed by incubation in the 3‐aminopropyl)triethoxysilane solution(1% w/w in 50% ethanol in deionized water) for 3 h at room temperature. The surface‐functionalized patch was thoroughly washed with isopropyl alcohol and dried under nitrogen flow before use. The change of the patch before and after treatment with functionalization solution was characterized by contact angle measuring instrument. The hydrogel pre‐solution was poured onto the surface‐functionalized patch, which was then cured with UV.

### 4.5 Mechanical Testing

The PGT hydrogels were synthesized with a rectangular shape (length = 25 mm, width = 10 mm, height = 1 mm). For the mechanical testing machine to grasp the end of the hydrogel, the grasping part of the front and backside of the PG hydrogel was bonded to a rigid polyethylene terephthalate (PET) film with super glue. Tensile mechanical tests were performed by using a universal testing machine with a loading rate of 100 mm/min. Young's modulus is further calculated from the average slope of the strain–stress curve(strain 2%–10%).

### 4.6 Peeling Test

Porcine skin was obtained from a local store. Adhesion tests proceeded with a 180°peeling test. PGT hydrogels were cut into a rectangular shape (length = 4 cm, width = 1 cm, and height = 1 mm) and were attached to the porcine skin with a similar size. A universal testing machine was used to record the force and stroke while the samples were applied with unidirectional tension at the loading rate of 10 mm/min. Interfacial toughness was calculated by dividing the plateau force two times by the width of the tissue sample following the corresponding ASTM standard.

### 4.7 Rheological Testing

The rheological behavior of hydrogels was studied using a rotating rheometer. The hydrogel precursor was poured into a circular mold with a diameter of 2 cm. After optical crosslinking, the gel sample with a thickness of about 1 mm was formed. The size of different samples tended to be consistent with the thickness. The sample is placed on the rheometer sample table, the gap between the rotor and the sample is adjusted, and the amplitude scan test is performed in the shear strain range from 0.01% to 10 000% to determine the rheological change with the shear strain tube.

### 4.8 Scanning Electron Microscopy

The hydrogel and EGaIn particles were imaged by SEM. For hydrogel samples, different gel samples should first be frozen in a −80°C refrigerator and then dried in a freeze dryer for 48 h to get drygel. The dry samples were sprayed with gold and finally placed in a vacuum environment, and the cross section morphology was observed and imaged under a voltage of 5 kV. For EGaIn particles, samples were also deposited by a thin layer of gold using an ion sputter. The stretched samples were fixed by conductive tape.

### 4.9 FTIR Testing

In order to determine whether the PGT hydrogels were successfully synthesized, the samples were tested using FTIR spectrometers. The sample was recorded against the background of blank KBr particles to detect the functional groups of the new synthetic material.

### 4.10 Electrochemical Characterization

Electrochemical impedance spectroscopy of PGT‐EEP was conducted using an electrochemical workstation, with Ag/AgCl as the reference electrode and Pt as the counter electrode. The recording sites of the PGT‐EEP were immersed in PBS solution while the other end was connected with the electrochemical workstation.

### 4.11 Electrical Performance Testing

The prepared epicardial electronic patch strain sensor part was connected to a digital multimeter through conductive copper tape to collect the resistance change curve of the patch in real time during the stretching process.

### 4.12 Contact Angle Test

The degree of surface modification and surface wettability of PDMS are determined by measuring the static water contact angle (WCA) that varies over time at room temperature. A syringe (2 µL) was used to drop the liquid onto the surface of the PDMS and the test was conducted using a contact Angle measuring instrument (model: JC2000FM).

### 4.13 In Vivo Experiment

Animals were anesthetized with 3% inhaled isoflurane. Use sterile eye ointment after anesthesia and before shaving to minimize the risks of corneal irritation, dehydration and sensitization during the surgery. Before the operation begins, the depth of anesthesia was checked by monitoring the compression responses of the tail and toes. Then remove the chest hair. Tracheal intubation was performed, the animal was connected to a mechanical ventilator, and it lay on its back on a heated pad during the operation. The shaving area was prepared by applying Betadine (iodine tincture) and subsequently rinsing with 70% ethanol three times, with each contact time being at least 2 min. The heart is exposed through thoracotomy at the third or fourth intercostal space on the left side, and the pericardium is removed with fine tweezers. Subsequently, PGT‐EEP was attached to the ventricular surface and connected to the four‐channel electrophysiological instrument and digital multimeter through the FFC flexible connector to obtain the epicardial electrocardiogram and the resistance change curve graph. We regulated the heart rate of rats by injecting normal saline, epinephrine, and metoprolol through the tail vein. The above‐mentioned experiment was approved by the Beijing Laboratory Animal Industry Association. My identification number: 1 123 042 000 030.

### 4.14 Hemolysis Experiment

First, take fresh rat blood and add anticoagulant, then centrifuge at 3500 revolutions per minute for 30 min. Then wash the red blood cells three times with PBS, and centrifuge under the same conditions each time. Subsequently, the washed red blood cell suspension was diluted 10 times with PBS. Then, 200 µL was added respectively to 1 mL of pure water, PBS, and dispersions of different materials. After incubating at 37°C for 5 h, centrifuge at 3000 revolutions per minute and observe the condition of the solution. Finally, 100 µL of the supernatant was transferred to a 96‐well plate, and the absorbance (OD value) was measured at a wavelength of 541 nanometers using an microplate reader. The calculation formula for hemolysis rate is: (Experimental group OD‐negative OD)/(Positive OD‐negative OD) ×100%.

### 4.15 Biocompatibility Test

PGT, PDMS, and EEP were washed three times with Phosphate Buffered Saline (PBS, ThermoFisher, USA), then sterilized in an ultraviolet environment, and subsequently the PGT, PDMS, and EEP extracts were prepared. We cultured fibroblast cells (3T3) and cardiomyocytes (HL‐1) in the extract and basal medium (37°C, 5% CO_2_). After culturing for 1, 3, 5, and 7 days, the cell proliferation was measured using a Cell Counting Kit‐8 (CCK‐8) assay. The calculation formula for cell viability is: (OD value of experimental group – OD value of the blank group)/(OD value of control group – OD value of the blank group) ×100%. On the other hand, live/dead staining were also carried out to observe cell morphology. Images of the unstained/stained cells were collected with an inverted phase‐contrast microscope. Furthermore, we also used the specific antibody staining method to identify cardiomyocytes and collected images. Similarly, to prove the compatibility in vivo, the PGT_10_ hydrogel and the patch were washed three times with PBS and then sterilized in a UV environment. Subsequently, it was implanted subcutaneously on the back of SD rats. Tissue sections were performed on the 7th and 14th days respectively, and H&E staining was used.

### 4.16 Statistical Analysis

All results were presented as the mean ± standard deviation (S.D.). All experiments were repeated for at least three times and each condition was analyzed in triplicate. One‐way analysis of variance (One‐way ANOVA) was used followed by Tukey's post‐hoc test to compare multiple groups, and the significance level was considered as ^*^ *p* < 0.05, ^**^ *P* < 0.01, ^***^ *P* < 0.001. All statistical analyses were carried out with the Origin 8.0.

## Conflicts of Interest

The authors declare no conflicts of interest.

## Supporting information




**Supporting File 1**: advs75098‐sup‐0001‐Supinfo.docx.

**Supporting File 2**: advs75098‐sup‐0002‐MovieS1.mp4.

## Data Availability

The data that support the findings of this study are available from the corresponding author upon reasonable request.
