# Supplementary material for: Soft and Adhesive Cardiac Patch for Electrophysiological and Contraction Measurement
Source: Adv Sci (Weinh). 2026 Apr 7;13(34):e75098. doi: 10.1002/advs.75098 (PMC13285110; doi:10.1002/advs.75098)
Supplement: Supplementary file 1 — Supporting File 1: advs75098‐sup‐0001‐Supinfo.docx. [file ADVS-13-e75098-s002.docx]

**Supplementary Information for**

**Soft and adhesive cardiac patch for electrophysiological and contraction measurement**

*Wuliang Chen^1#^, Xinyu Shen^2#^, Hanning Liu^3#^, Lamei Du^4^, Caicai Jiao^1^, Longfei Li^5^, Qian Wang^1^, Qiuting Zhang^4^, Lixue Tang^2*^, Liang Hu^1*^, Yubo Fan^1*^*

1. School of Biological Science and Medical Engineering, Beihang University, Beijing, 100191, China

2. School of Biomedical Engineering Capital Medical University, Beijing, 100069, China.

3. National Center for Cardiovascular Diseases, National Clinical Research Center for Cardiovascular Diseases, State Key Laboratory of Cardiovascular Disease, Department of Cardiovascular Surgery, Fuwai Hospital, Key Laboratory of Coronary Heart Disease Risk Prediction and Precision Therapy, Chinese Academy of Medical Sciences and Peking Union Medical College, Beijing 100037, China.

4. School of Mechanical Engineering and Automation, Beihang University, Beijing, 100191, China

5. College of Automation Engineering, Northeast Electric Power University, Jilin 132012, P. R. China.

# These authors contribute equal to this work.

*Corresponding author: [cnhuliang@buaa.edu.cn](mailto:cnhuliang@buaa.edu.cn), [yubofan@buaa.edu.cn](mailto:yubofan@buaa.edu.cn), [tanglx@ccmu.edu.cn](mailto:tanglx@ccmu.edu.cn).


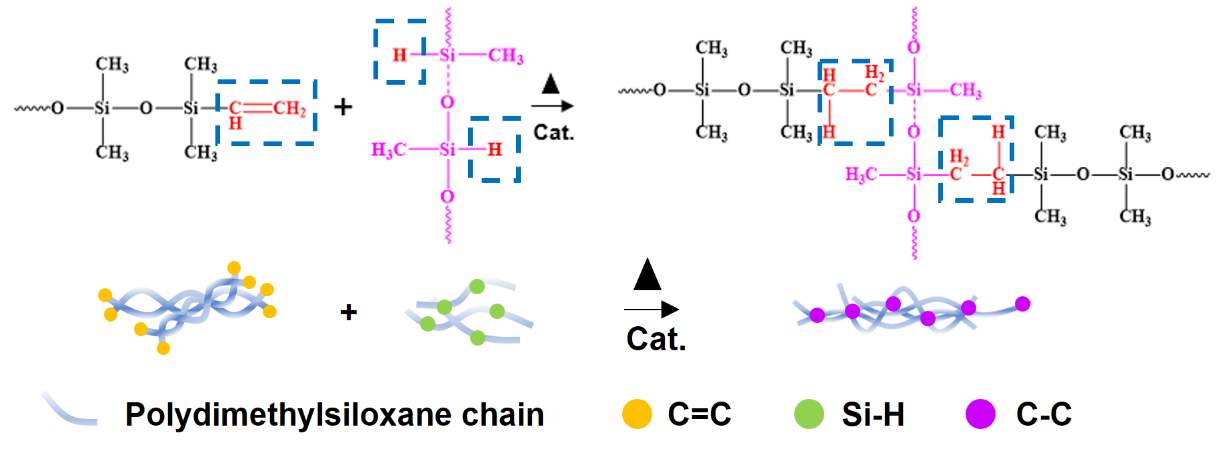


Figure S1. The crosslinking mechanism of PDMS. Under platinum catalysis, dimethylsiloxane oligomers with vinyl-terminal groups and curing agents containing Si-H bonds polymerize to form a network, in which heating can accelerate crosslinking.


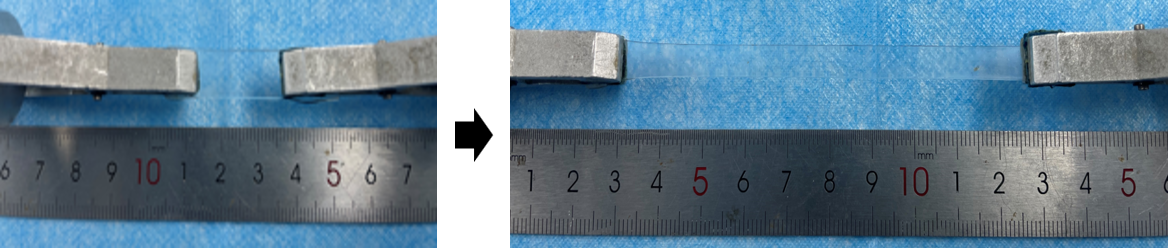


Figure S2. The stretchability of PGT_10_ hydrogel


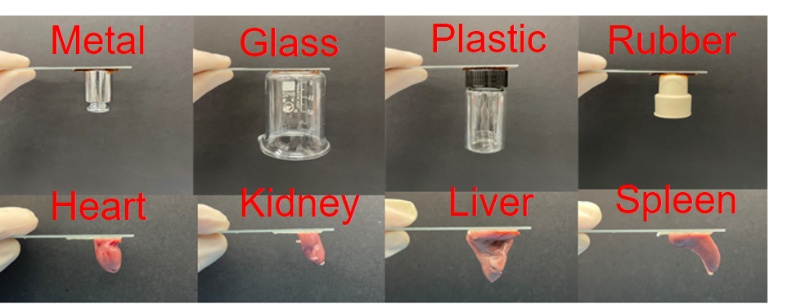


Figure S3. The adhesion characteristics of PGT_10_ hydrogels to different materials.


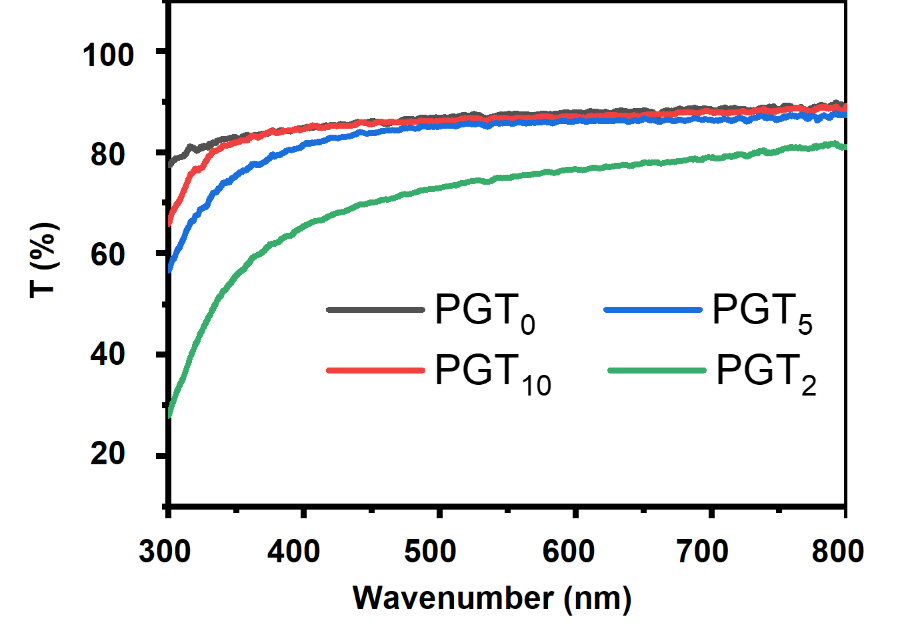


Figure S4. The transparency of hydrogels with different ratios of acrylamide to gelatin.


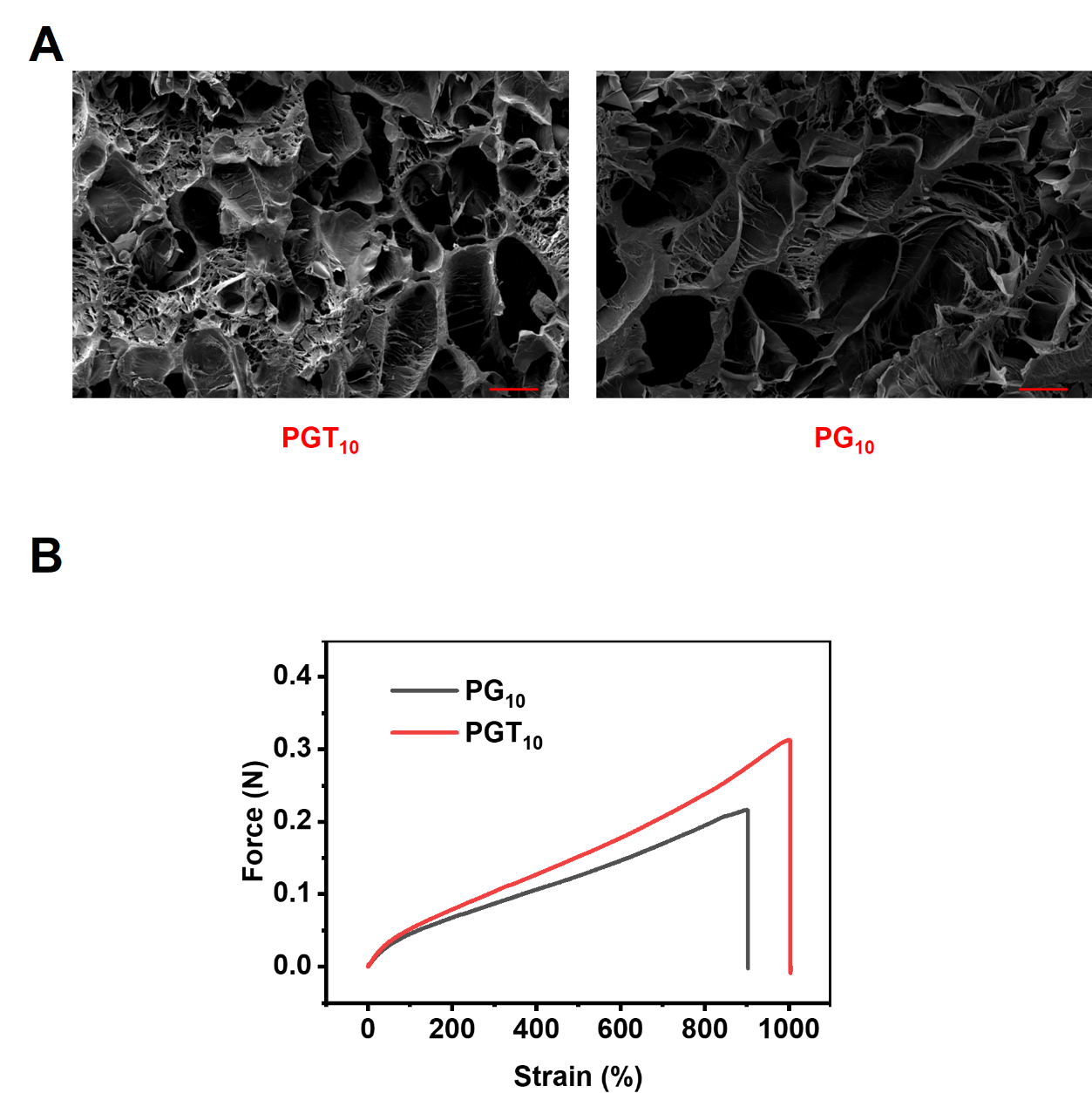


Figure S5. A) SEM images of PG_10_ hydrogel and PGT_10_ hydrogel; B) Tensile characteristic curves of PG_10_ hydrogel and PGT_10_ hydrogel.


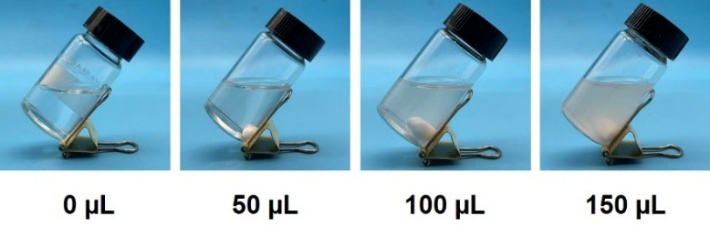


Figure S6. Images of precursor solutions with different tannic acid contents.


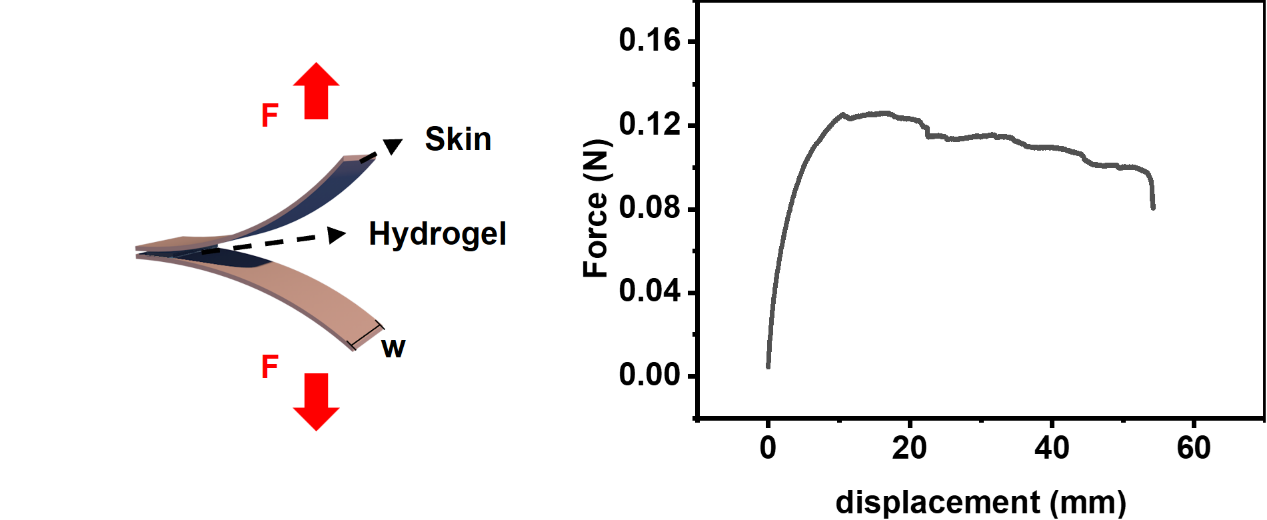


Figure S7. Adhesion force test of hydrogels without gelatin molecules.


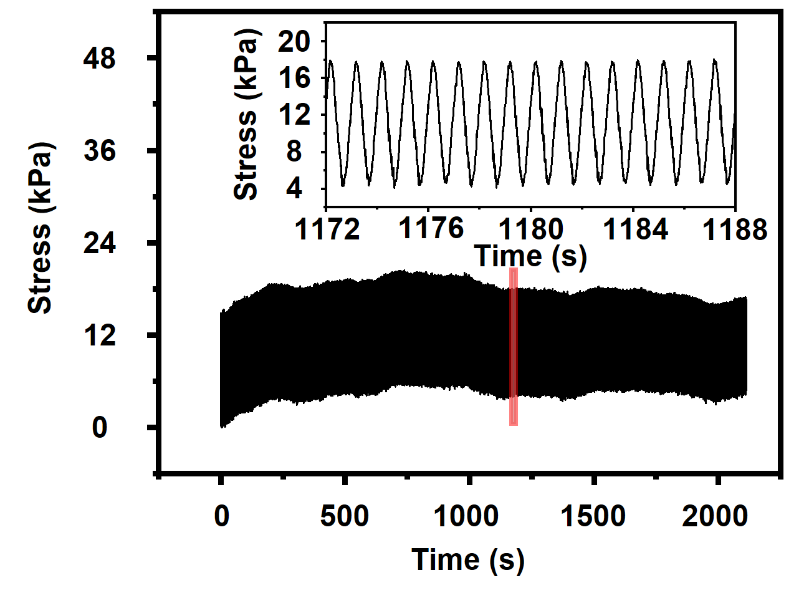


Figure S8. The adhesion force of the PGT_10_ to porcine skin remains stable under cyclic tensile loading (0–10% strain, 2000 cycles).


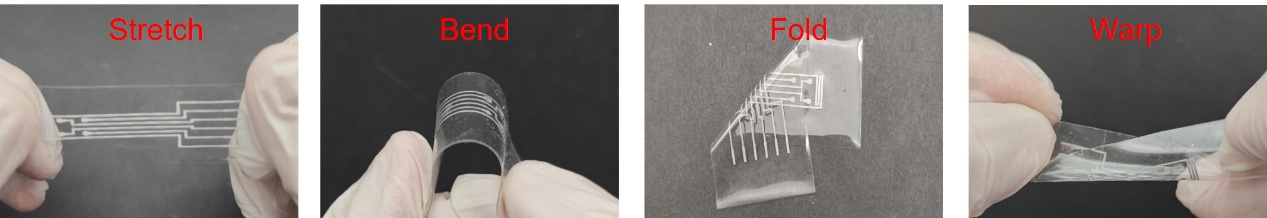


Figure S9. The epicardial electronic patch has a high degree of flexibility and can be stretched, bent, folded and warp.


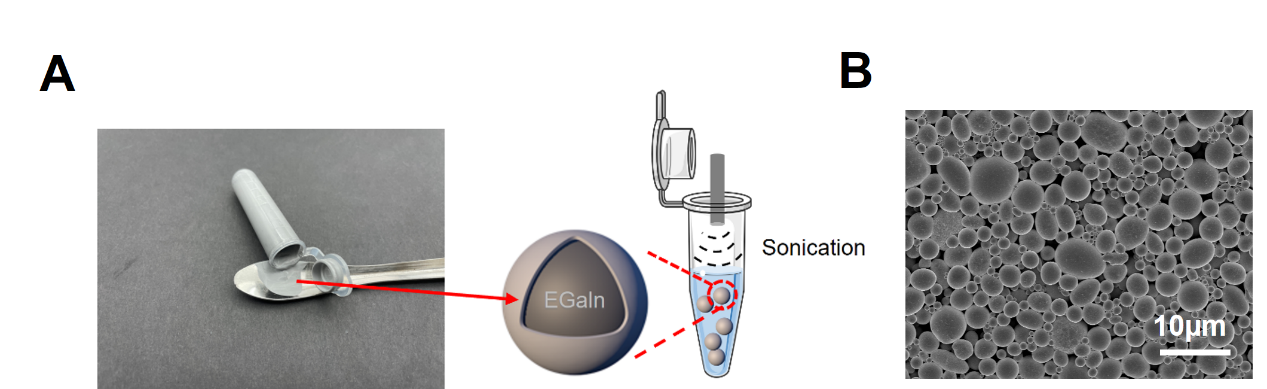


Figure S10. A) Liquid metal paste for screen printing and its preparation principle. B) The microstructure of liquid metal particles, with a size of approximately 2.5 µm.


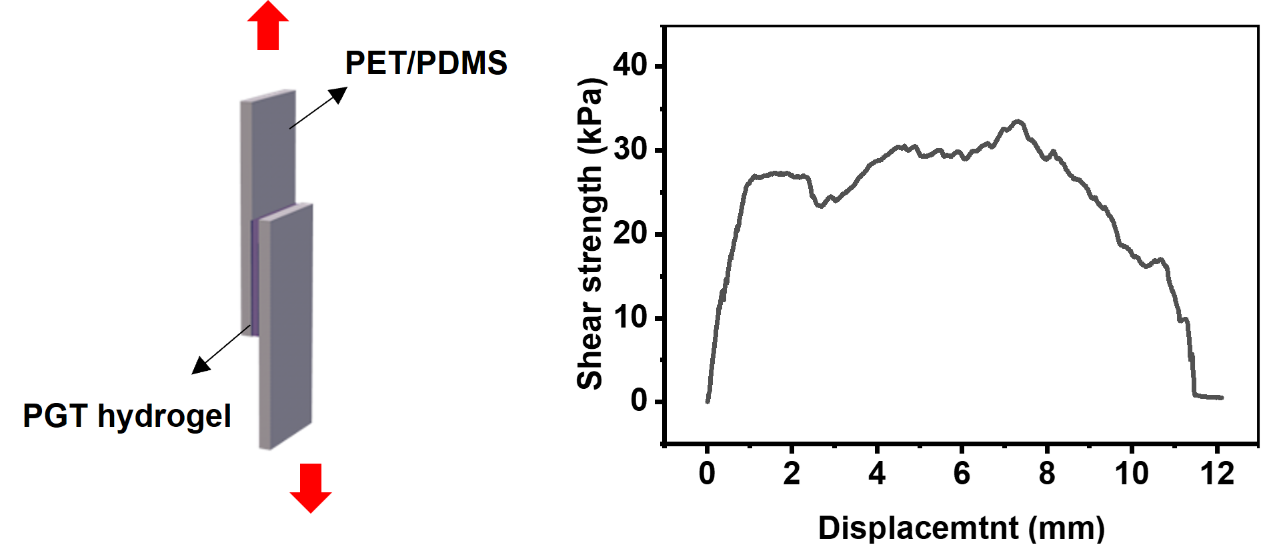


Figure S11. Binding force between hydrogel and PDMS. Schematic illustration of the lap-shear test, and representative shearing curves of adherent joints between PDMS samples and PGT_10_ hydrogels.


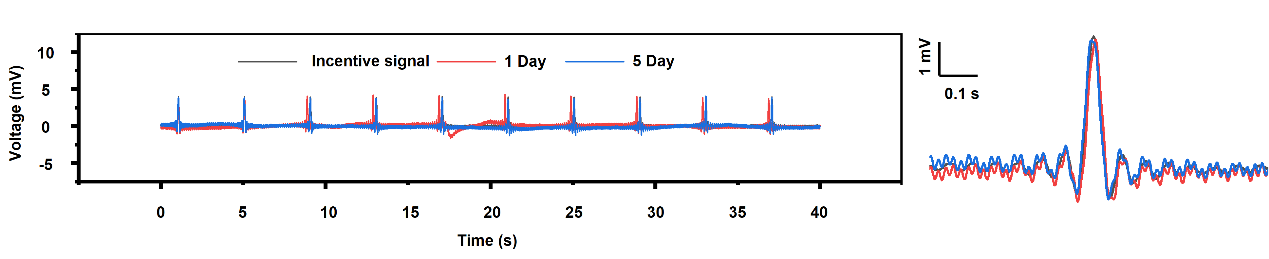


Figure S12. Comparison of signals collected before soaking and 5 days after soaking.


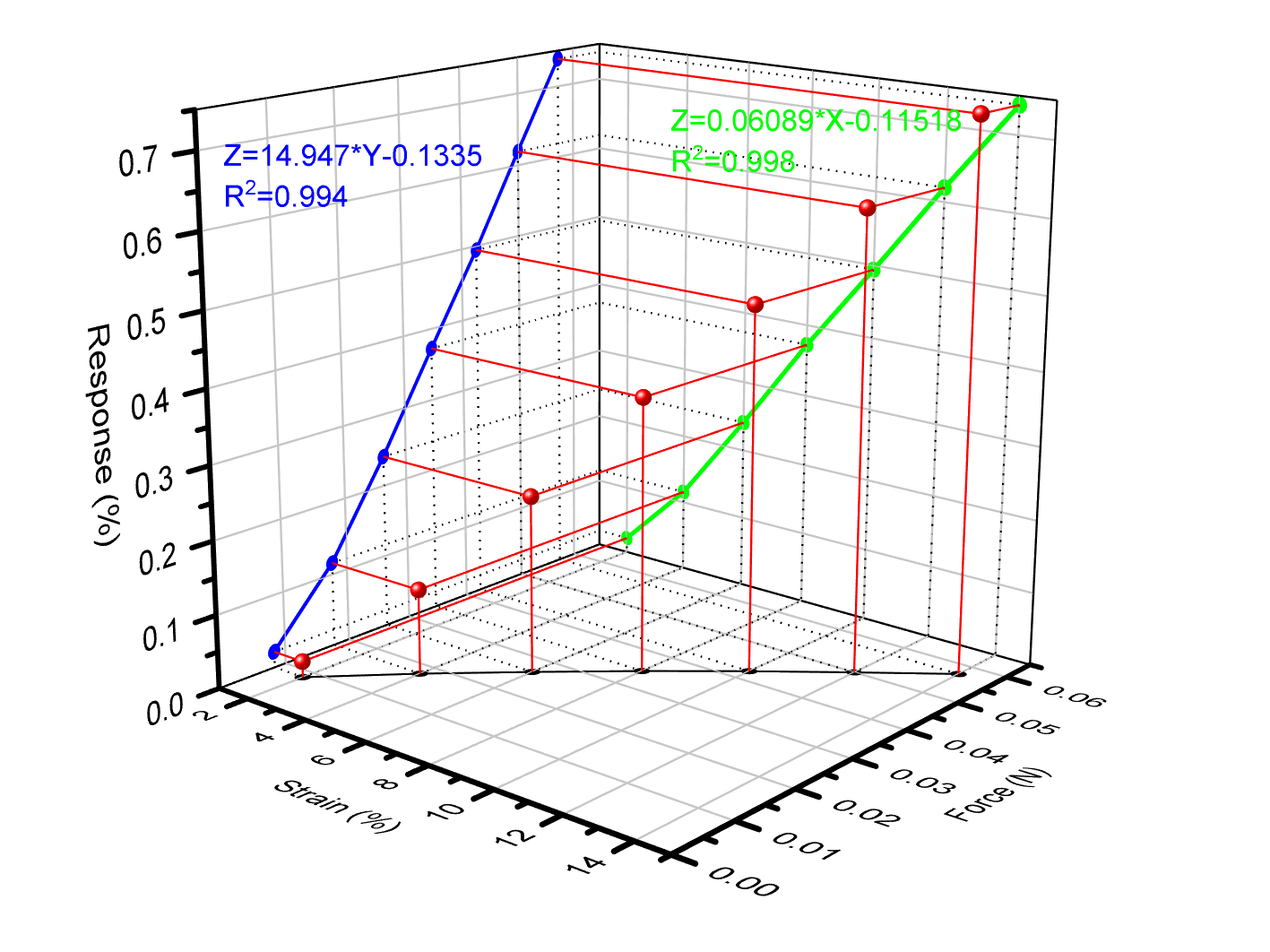


Figure S13. The corresponding relationship between strain (X-axis), stress (Y-axis), and sensitivity (Z-axis).


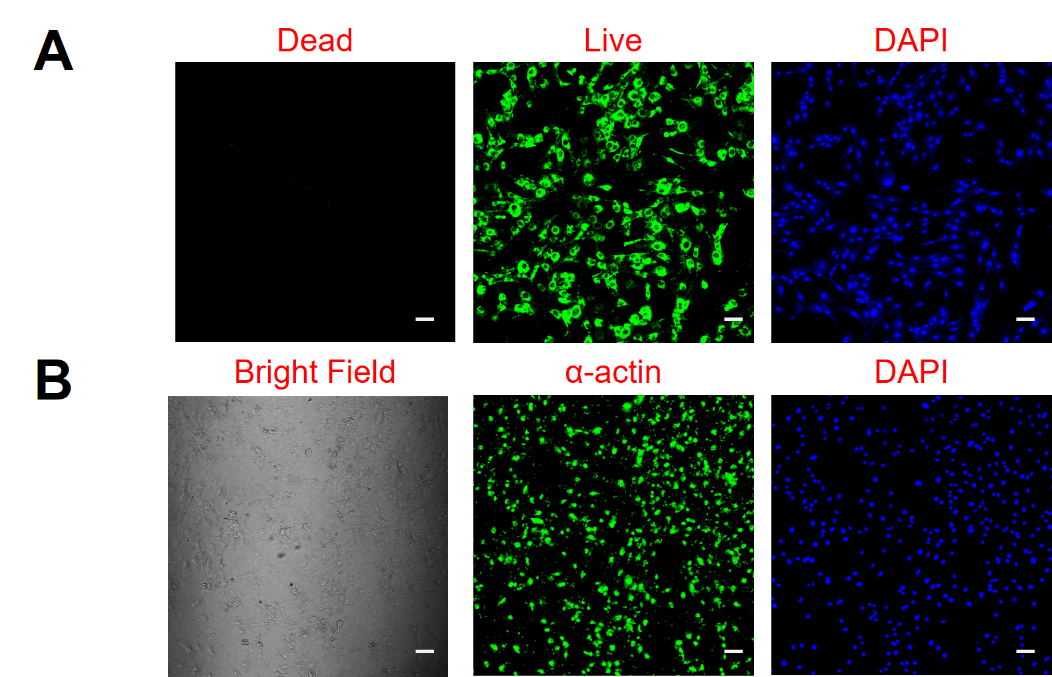


Figure S14. A) Fluorescence images of cardiomyocytes co-cultured with PGT-EEP for 3 days, green (calcium yellow green acetyl methyl ester, Calcein-AM), red (PI), with a scale size of 100 μm. B) Fluorescence images of cardiomyocytes, green (α -actin), blue (DAPI, 4',6-diamino-2-phenylindole), bright field; The scale size is 100 μm.

Table S1. A summary of electronic devices.

|  | Maximum strain | | Modulus | | Adhesion force | Animal | Conductor | Design | Ref. |
| --- | --- | --- | --- | --- | --- | --- | --- | --- | --- |
| Nancomposite cadiac mesh | 100% | 1-2MPa | | Non-adhesion | | Porcine | Ag-AuNW | MEMS | ^[S1]^ |
| Elastic electrode array | 20% | NA | | Non-adhesion | | Porcine | Au/PEDOT:PSS | MEMS | ^[S2]^ |
| Rubbery patch | 30% | 1MPa | | Non-adhesion | | Porcine | AgNW/PDMS | MEMS | ^[S3]^ |
| Stretchable electronic patch | 40% | 1.3MPa | | Non-adhesion | | Bullfrog&Rabbit | EGaln | MEMS | ^[S4]^ |
| Ultraflexible multielectrode arrays | 15% | ~10^3^MPa | | NA | | Rat | Au | MEMS | ^[S5]^ |
| Transistor array | NA | NA | | Non-adhesion | | Rabbit | Cr/Au | MEMS | ^[S6]^ |
| Stretchable electronic patch | 660% | 528.5kPa | | 7.2kPa | | Rat | EGaln | Electrospinning | ^[S7]^ |
| Electrode Arrays | 0.8%-1.4% | ~10^3^MPa | | Non-adhesion | | Rabbit& Rat | Al | MEMS | ^[S8]^ |
| Stretchable  Bioelectrode Arrays | 100% | NA | | Non-adhesion | | Rat | PPy electrode | MEMS | ^[S9]^ |
| Hydrogel Bioelectronics | 375% | ~600kPa | | ~100kPa | | Rat | PEDOT:PSS | 3D printing | ^[S10]^ |
| Electrode Array | 30% | 10MPa | | Non-adhesion | | Rat | Mo | MEMS | ^[S11]^ |
| electromyography electrode array patch | 188% | 644kPa | | 0.58N/cm | | NA | PEDOT:PSS | Screen printing | ^[S12]^ |
| Flexible electrodes | 70% | 1.63MPa | | Non-adhesion | | NA | Au NWs- PPy | Electrochemical/ MEMS | ^[S13]^ |
| Electrode array | ~100% | 18.1-28.7 MPa | | Non-adhesion | | Rat&  monkeys | Au | MEMS | ^[S14]^ |
| Stretchable electrode array | 260% | 65kPa | | 12kPa | | Rat | EGaln | Screen printing | This work |

**References：**

[1] F. Xu, Y. Zhu, *Advanced Materials* **2012**, *24*, 5117.

[2] J. Liu, X. Zhang, Y. Liu, M. Rodrigo, P. D. Loftus, J. Aparicio-Valenzuela, J. Zheng, T. Pong, K. J. Cyr, M. Babakhanian, J. Hasi, J. Li, Y. Jiang, C. J. Kenney, P. J. Wang, A. M. Lee, Z. Bao, *Proc. Natl. Acad. Sci. U.S.A.* **2020**, *117*, 14769.

[3] K. Sim, F. Ershad, Y. Zhang, P. Yang, H. Shim, Z. Rao, Y. Lu, A. Thukral, A. Elgalad, Y. Xi, B. Tian, D. A. Taylor, C. Yu, *Nat Electron* **2020**, *3*, 775.

[4] S. Wang, Y. Nie, H. Zhu, Y. Xu, S. Cao, J. Zhang, Y. Li, J. Wang, X. Ning, D. Kong, *Sci. Adv.* **2022**, *8*, eabl5511.

[5] W. Lee, S. Kobayashi, M. Nagase, Y. Jimbo, I. Saito, Y. Inoue, T. Yambe, M. Sekino, G. G. Malliaras, T. Yokota, M. Tanaka, T. Someya, *Sci. Adv.* **2018**, *4*, eaau2426.

[6] J. C. Hwang, M. Kim, S. Kim, H. Seo, S. An, E. H. Jang, S. Y. Han, M. J. Kim, N. K. Kim, S.-W. Cho, S. Lee, J.-U. Park, *Sci. Adv.* **2022**, *8*, eabq0897.

[7] H. Choi, Y. Kim, S. Kim, H. Jung, S. Lee, K. Kim, H.-S. Han, J. Y. Kim, M. Shin, D. Son, *Nat Electron* **2023**, *6*, 779.

[8] T. A. Truong, X. Huang, M. Barton, A. Ashok, A. Al Abed, R. Almasri, M. N. Shivdasanic, R. Reshamwala, J. Ingles, M. T. Thai, C. C. Nguyen, S. Zhao, X. Zhang, Z. Gu, A. Vasanth, S. Peng, T.-K. Nguyen, N. Do, N.-T. Nguyen, H. Zhao, H.-P. Phan, *ACS Nano* **2025**, *19*, 1642.

[9] Q. Zhao, M. Zhu, G. Tian, C. Liang, Z. Liu, J. Huang, Q. Y. Yu, S. Tang, J. Chen, X. Zhao, Q. Zeng, C. Guo, D. Qi, *Adv Healthcare Materials* **2023**, *12*, 2203344.

[10] F. Wang, Y. Xue, X. Chen, P. Zhang, L. Shan, Q. Duan, J. Xing, Y. Lan, B. Lu, J. Liu, *Adv Funct Materials* **2024**, *34*, 2314471.

[11] K. Xu, S. Li, S. Dong, S. Zhang, G. Pan, G. Wang, L. Shi, W. Guo, C. Yu, J. Luo, *Adv Healthcare Materials* **2019**, *8*, 1801649.

[12] S. Yang, J. Cheng, J. Shang, C. Hang, J. Qi, L. Zhong, Q. Rao, L. He, C. Liu, L. Ding, M. Zhang, S. Chakrabarty, X. Jiang, *Nat Commun* **2023**, *14*, 6494.

[13] H. Zhang, Y. Shen, L. Xing, W. Wang, L. Liu, R. Jin, Z. He, Y. Wang, S. Yao, X. Cao, S. Dong, B. Zhu, *Chemical Engineering Journal* **2025**, *508*, 160973.

[14] H. Moon, J.-W. Jang, S. Park, J.-H. Kim, J. S. Kim, S. Kim, *Sensors and Actuators B: Chemical* **2024**, *401*, 135099.
